# The prevalence of horse trypanosomiasis in Sumba Island, Indonesia and its detection using card agglutination tests

**DOI:** 10.14202/vetworld.2019.646-652

**Published:** 2019-05-09

**Authors:** Wisnu Nurcahyo, Marlin R. K. Yowi, Sri Hartati, Joko Prastowo

**Affiliations:** 1 Department of Parasitology, Faculty of Veterinary Medicine, Universitas Gadjah Mada, Karangmalang, Yogyakarta 55281, Indonesia; 2 Department of Animal Health, Polytechnic of Agriculture Kupang, Jalan Prof. Herman Yohannes Lasiana, Kupang, Nusa Tenggara Timur, Indonesia; 3 Department of Internal Medicine, Faculty of Veterinary Medicine, Universitas Gadjah Mada, Karangmalang, Yogyakarta, Indonesia

**Keywords:** CATT, horses, Sumba Island, *Trypanosoma evansi*

## Abstract

**Background and Aim:**

Horses have a strategic and vital role to play in the lives of the people of Sumba Island, East Nusa Tenggara Province. They act as social animals that are involved in death ceremonies, horse races, and during pasola, thereby supporting tourism, and are given away as dowry in wedding ceremonies. This study aimed to investigate the prevalence of trypanosomiasis among horses in four districts of Sumba Island by examining clinical symptoms and detecting parasites, antibodies, and other factors that are related to *Trypanosoma evansi* infection in horses.

**Materials and Methods:**

We studied a total of 211 horses that belonged to 88 clinical hobby breeders. Giemsa-colored smears and serum were examined in order to detect antibodies using card-agglutination tests (CATT). The study was conducted during the rainy season that lasted from January to March 2017. Potential risk factors such as the species, sex, origin of the livestock, how the livestock were maintained, and the farmers’ knowledge concerning trypanosomiasis were recorded using questionnaires. Data were collected annually for three years from 2010-2012 and repeatedly analyzed by a Chi-square test.

**Results:**

Clinical signs of trypanosomiasis were found in 34 horses; blood smears were examined using Giemsa staining and negative preparations were obtained at a frequency of 0.0% (0/211). The CATT results generally showed that 13.3% (28/211) of the samples were seropositive for antibodies to *T. evansi*; the highest percentage, 16.67% (8/48), of seropositivity was found in the West Sumba District, and the lowest, 12.0% (5/50), was found in Southwest Sumba. The incidence of trypanosomiasis was higher (75% [21/28]) among female hip horses; horses with 1-5 years of experience were more susceptible to a *T. evansi* infection (46.4% [13/28]). In general, farmers on Sumba Island knew of trypanosomiasis (89.8% [79/88]), and 69.3% (61/88) of the farmers reported that their livestock was sick. This study was the first serological study conducted on trypanosomiasis in horses of Sumba Island after the surra outbreak in 2010-2012. There were 3% of farmers who were willing to provide the government with information on implementing a prevention program and controlling the spread of surra on the island.

**Conclusion:**

The diagnoses of surra disease were made based on clinical symptoms and parasitological examinations. CATTs could be used to diagnose *T. evansi* infection in horses.

## Introduction

Horses have a strategic and vital role to play in the lives of the people of Sumba Island, East Nusa Tenggara Province. They act as social animals that are involved in death ceremonies, horse races, and pasola, thereby supporting tourism, and are given away as dowry during wedding ceremonies. Its vast savannah grassland makes Sumba Island a potential area in Indonesia for breeding livestock, such as horses and cattle. The surra outbreak in Sumba Island occurred from 2010 to 2012, and the outbreak affected 4268 animals, out of which 1159 animals were horses [1].

Surra is a disease that is caused by *Trypanosoma evansi*. The disease is almost always fatal to horses. If affected horses are untreated, they can die within 1 week to 6 months. *T. evansi*-infected horses do not exhibit clinical symptoms [2,3]. *T. evansi* infections are classified into two types: acute and chronic infections. Typically, the infection lasts for 3 months to 3 years. The clinical symptoms exhibited by animals with trypanosomiasis include urticaria, hemorrhaging in the mucosal membrane, intermittent fever, anemia, edema, icterus, swollen lymph nodes, weakness, paralysis, anorexia, miscarriage, digestive disorders, conjunctivitis, pink eye, hair loss, and body weight loss [2,4,5]. The variation in the incidence of surra depends on the sensitivity of animals to the infection and to the presence of host vectors such as the *Tabanus* and *Stomoxys* spp. The infection causes a significant decrease in reproduction and body weight and an increase in anemia and abortus among domestic Asian, African, and South American species [5].

This study on the prevalence of surra will provide the government with information on implementing a prevention program and controlling the spread of surra on Sumba Island.

This study aimed to investigate the prevalence of trypanosomiasis among horses from four districts of Sumba Island by examining the associated clinical symptoms and by detecting the parasites, antibodies, and other factors related to *T. evansi* infections in horses.

## Materials and Methods

### Ethical approval

The approval to conduct this study was obtained via the following permissions: permission from the livestock agency of West Sumba with the approval number, 191/K.10/TU/PP/12/2017; a letter of recommendation from Bappeda of Southwest Sumba with the ID, 222/BP.03/12/2017; permission from the agriculture agency of Middle Sumba with the SK number, 188/PP/TU/12/2017; and permission from the agriculture and livestock agency of East Sumba with the SK number, 246/Dinpet/TU/12/2017.

### Study areas

The study was conducted in four districts of Sumba Island, namely West Sumba, Southwest Sumba, Middle Sumba, and East Sumba. The astronomic location of the island is 118º 55’-120º 52’ east longitude and 09º 16’-10º 20’ south latitude. The Sumba island is 1,105,242 km^2^ wide and has a dry climate (semiarid to arid). The districts of Middle Sumba, West Sumba, and Southwest Sumba are wetter than East Sumba. The rainy season is short and lasts for 3–5 months (January–March or December–April) in East Sumba and 4–5 months (December/January-April) in West Sumba.

### Research design and animal samples

Samples were drawn from horses that were raised extensively and semi-extensively in the period between January to June 2017 using a simple, random sampling technique. The samples were drawn Please define P, Q, and L.for three replications of the tests. The sample size was calculated using the estimated prevalence with the formula: 4PQ/L^2^. P = Prevalence; Q = 100-P; L = 15% of P. The prevalence design was based on the prevalence of surra among cattle that was reported on by the Provincial Livestock Service, East Nusa Tenggara, which was 10.28% out of 152 samples. The samples were assigned to age groups of below 1 year, 1-5 years, and over 5 years. Data, including the sex, age, origin of the livestock, raising system, farmers’ knowledge on surra, and awareness of the farmers in terms of reporting a sick animal to a veterinarian, were collected using questionnaires. Data on clinical symptoms related to *T. evansi* infection, including the presence of anemia, body weight loss, neurological disorders such as paralysis, and extremity edema or testicular edema, were collected during sampling.

### Specimen collection

The blood samples were collected from the jugular vein of each animal using a syringe, and they were then stored in vacutainer tubes without heparin. Slanted glass tubes containing blood samples were used to obtain serum. Blood from a syringe was dropped onto an object glass to prepare blood smear slides. Moreover, following this, the slides were dried and fixed using methanol for 3 min and were then tinted using Giemsa for the parasitological examination. The serum was moved into an Eppendorf tube and stored at −20°C until the antibodies were examined according to the procedure recommended by the Institute of Tropical Medicine, Antwerp, Belgium. At pH 7.2, 20 µl serum was diluted in 60 µl phosphate buffer saline using an enzyme-linked immunosorbent assay plate and then homogenized using a “shaker.” Approximately, 45 µl of the solution was dropped directly onto the circular card-agglutination test (CATT) cards, and 25 µl serum was added and homogenized using a stirrer. Each CATT card was placed on a rotator; the rotator was switched on at a velocity of 70 rpm for 5 min, and the result was read on a visual display. The specimen was considered positive when the agglutinate turned blue [6].

### Statistical analysis

The prevalence of the disease was estimated by dividing the number of positive samples detected via the CATT by the total number of samples collected from the horses. The prevalence percentage was calculated for Sumba Island as a whole and for each of the districts individually. The risk factors that were associated with surra were reported on descriptively for the entire island and for each district. Data were collected annually via observations conducted between 2010 and 2012 and were analyzed using Chi-square test.

## Results

**Figure-1:**
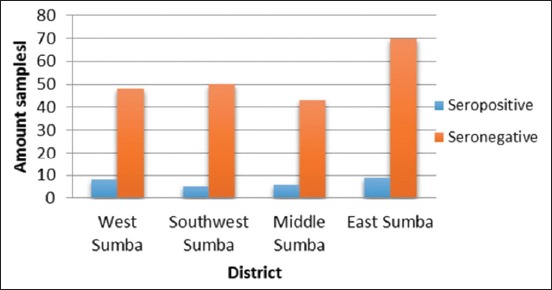
The histogram representing CATT results in four districts of Sumba.

The clinical symptoms of surra include anemia, body weight loss, neurological disorders such as paralysis, and extremity edema or edema testes (not all the clinical symptoms were found in a single animal). Results of all the blood samples were negative. The highest percentage of seropositive results indicating the presence of surra was found in the West Sumba District, and the lowest was found in the Southwest Sumba District (Figure 1). During the study, the animals were found to be in a poor and seronegative condition, and they did not exhibit any of the clinical symptoms associated with surra.

Based on the results of the CATTs conducted on horses from Sumba Island (Table-1), the number of seronegative horses was larger than that of seropositive horses in all the districts of Sumba Island (p<0.05).

**Table-1:** CATT results on the prevalence of trypanosomiasis prevalence in four districts of Sumba.

Group	Seronegative (%)	Seropositive (%)	Chi-square analysis
Southwest Sumba	90	10	Χ^2^ result=1.98
West Sumba	83.30	16.70	Χ^2^ table=7.81
East Sumba	87.1	12.9	Χ^2^ result <Χ^2^ table
Middle Sumba	86.1	13.9	(p>0.05)

Based on the results presented in Table 1, it can be established that there were no significant differences observed in the results of the serological examinations and that the total number of seropositive horses was smaller than that of seronegative horses. The difference was in the sampling location.

**Figure-2:**
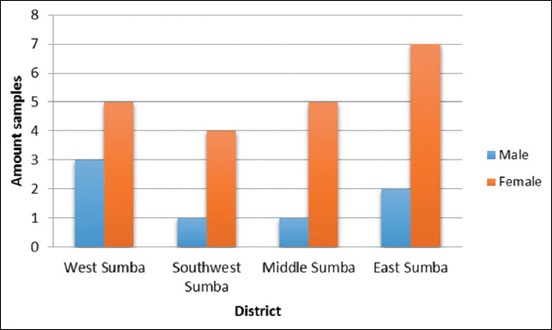
The histogram representing the CATT results based on sex.

According to the CATT results based on the sex of the horses, it was found that the seropositive percentage among the female horses was higher than that among the male horses in all the districts of Sumba Island (p<0.05). The results of the analysis conducted on the risk factors associated with the surra disease are summarized in Figure-2 and Table-2.

**Table-2:** CATT results on the prevalence of trypanosomiasis based on sex.

Group	Male (%)	Female (%)	Chi-square analysis
Southwest Sumba	20	80	Χ^2^ result=13.92
West Sumba	37.5	62	Χ^2^ table=7.81
East Sumba	22.2	77.8	Χ^2^ result >Χ^2^ table
Middle Sumba	16.7	83.3	(p<0.05)

It is evident from the results presented in Table-2 that there was a significant difference in the serological examination results based on the sex of the animal and that the total number of female horses that were affected was higher than that of male horses. There were no significant differences observed in terms of the sampling location. Mares showed higher surra seropositivity than stallions in all the districts (Figure-2).

The results of the CATTs based on age demonstrated that the horses that were under 1 year old had a lower seropositive percentage than those that were 1-5 years old and those that were above 5 years old in all the districts in Sumba Island (p<0.05) (Table-3).

**Table-3:** The CATT results on trypanosomiasis prevalence based on age.

Group	<1 year	1-5 years	>5 years	Chi-square analysis
Southwest Sumba	25	50	25	Χ^2^ result=1.98
West Sumba	0	60	40	Χ^2^ table=7.81
East Sumba	0	16.7	83.3	Χ^2^ result <Χ^2^ table
Middle Sumba	11.12	55.55	33.33	(p>0.05)

**Figure-3:**
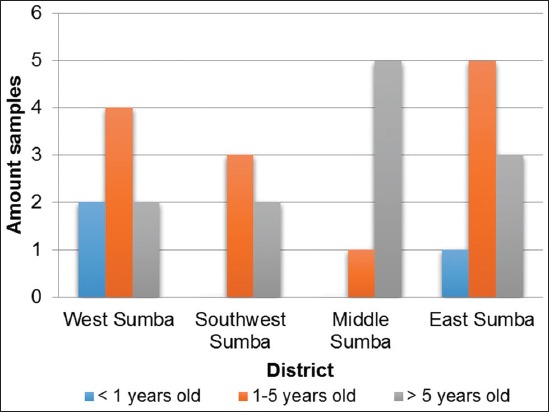
The histogram representing the CATT results based on horse age.

It can be clearly observed from the results presented in Table 3 and Figure 3 that the serological examination results based on the age of the animals did not result in significant differences; the total number of seropositive horses under the age of 1 year was lower than the number of those aged between 1–5 years. There were no significant differences in the results in terms of the sampling location.

In general, the adult and old horses were more likely to be more susceptible to a *T. evansi* infection than the young horses were (<1 year) (Figure-3).

The CATT results based on the animals’ place of origin showed that the horses that were bought from other locations had a higher seropositive percentage than those that were acquired from their own mothers places of origin, which were Southwest Sumba and West Sumba (p<0.05). On the contrary, in East and Middle Sumba, the seropositive percentage of the bought horses was lower than that of the horses from their own mothers place of origin (p<0.05) (Table-4).

**Table-4:** CATT trypanosimiasis results on based on the the animals’s origin.

Group	Horses acquired from their own mother (%)	Horses acquired via purchase (%)	Chi-square analysis
Southwest Sumba	47.16	52.84	Χ^2^ result=14.64
West Sumba	40	60	Χ^2^ table=7.81
East Sumba	57.15	42.85	Χ^2^ result >Χ^2^ table
Middle Sumba	65.11	34.89	(p<0.05)

Based on the data presented in Table 4, it can be established that the horses’ origins resulted in significant differences in the serological examination results and that the total number of seropositive horses that were from their own mothers’ place of origin was higher than that of those that were bought in a different place of origin. There were no significant differences based on the sampling location.

**Figure-4:**
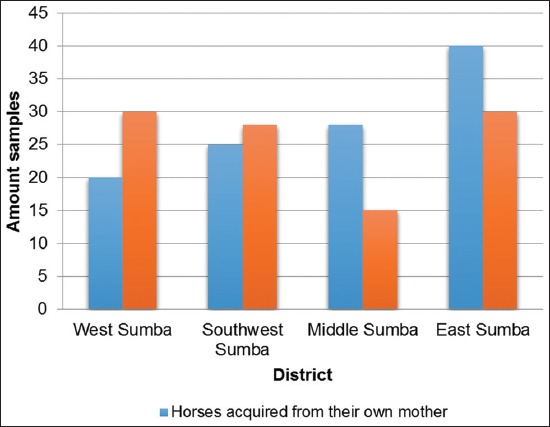
The histogram representing CATT results based on the horses’ origins.

The majority of the horses in East Nusa Tenggara came from their own mothers place of origin when compared to the horses that were bought from places outside the region. The horses that were from the same place of origin as their own mothers were more vulnerable than the horses that were bought for the purposes of dowry or gifts (Figure-4).

The CATT results based on the farming models showed that the seropositivity percentage of the non-pastured horses was higher than that of the pastured ones in all the districts of Sumba Island (p<0.05) (Table-5).

**Table-5:** The CATT results on trypanosomiasis baesd on farming models.

Group	Pastured (%)	Non-pastured (%)	Chi-square analysis
Southwest Sumba	20	80	Χ^2^ result=13.93
West Sumba	37.5	62.5	Χ^2^ table=7.81
East Sumba	22.2	77.8	Χ^2^ result >Χ^2^ table
Middle Sumba	16.7	83.3	(p<0.05)

Based on the data presented in Table-5, it can be established that the horse farm model resulted in significant differences in the serological examination results and that the total number of seropositive non-pastured horses is larger than the number of pastured ones. There were no significant differences based on the sampling location.

**Figure-5:**
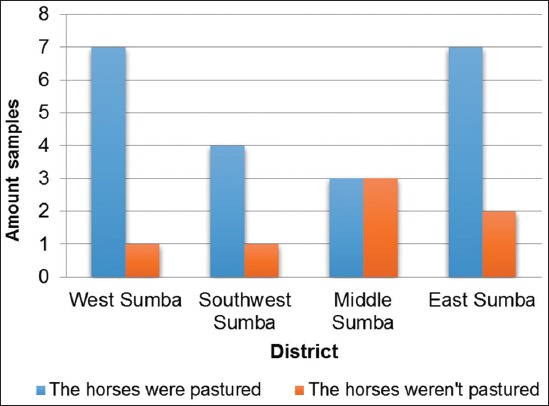
The Histogram representing the CATT results based on farming models.

There was a larger number of pastured horses in East Nusa Tenggara compared to the number of free-range horses. The pastured horses had higher seropositive percentages associated with surra compared to the free-range horses (Figure-5).

The CATT results based on the farmers’ knowledge of trypanosomiasis showed that the seropositivity percentage of the horses owned by farmers who knew about trypanosomiasis was higher than that of the horses owned by farmers who did not know about trypanosomiasis in Southwest Sumba, West Sumba, and East Sumba (p<0.05). On the contrary, the seropositivity percentage of the horses owned by farmers who knew about trypanosomiasis was lower than that of the horses owned by farmers who did not know about trypanosomiasis in Middle Sumba (p<0.05) (Table-6).

**Table-6:** CATT results on trypanosomiasis based on farmer’s knowledge.

Group	Farmers knew about trypanosomiasis (%)	Farmers did not know about trypanosomiasis (%)	Chi-square analysis
Southwest Sumba	62.10	37.90	Χ^2^ result=224.38
West Sumba	95	5	Χ^2^ table=7.81
East Sumba	100	0	Χ^2^ result >Χ^2^ table
Middle Sumba	11.80	88.2	(p<0.05)

Based on an ANOVA analysis (Table-6), it can be established that the farmer’s knowledge resulted in a significant difference in the serological examination results and that the total number of farmers who knew about trypanosomiasis was larger than that of farmers who did not know about trypanosomiasis. There were no significant differences based on the sampling location.

**Figure-6:**
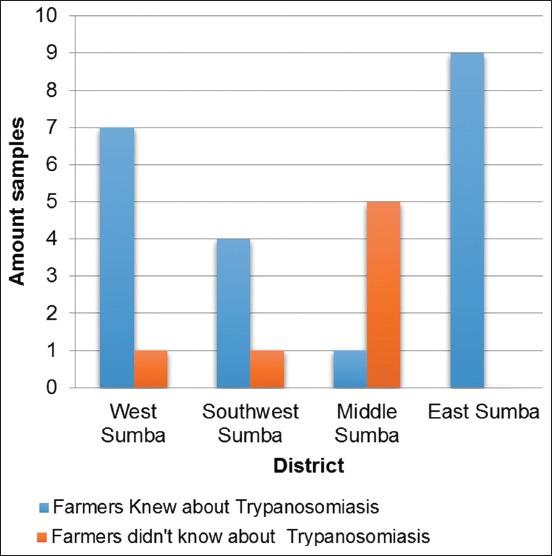
Histogram of CATT results based on farmer trypanposomiasis knowledge.

Among the farmers from the four districts, the farmers from East Sumba had the most knowledge on surra (Figure-6). Horses that were raised by farmers with a higher knowledge level exhibited lower seropositive surra percentages than those raised by farmers with a lower knowledge level.

Data on the willingness of farmers to report their sick animals showed that the seropositivity percentage of horses owned by breeders who reported their sick animals was higher than that of the horses owned by farmers who did not report their sick animals in all the districts in Sumba (p<0.05) (Table-7).

**Table-7:** CATT results based on the farmer’s willingness to report sick animals.

Group	Farmers who reported their sick animals (%)	Farmers who did not report their sick animals (%)	Chi-square analysis
Southwest Sumba	82.80	17.20	Χ^2^ result=12.54
West Sumba	95	5	Χ^2^ table=7.81
East Sumba	95.45	4.55	Χ^2^ result >Χ^2^ table
Middle Sumba	88.20	11.8	(p<0.05)

Based on an ANOVA analysis (Table-7), it can be established that the farmers’ awareness in terms of reporting the onset of the disease resulted in significant differences in the serological examination results of their animals; the total number of the farmers who reported that their animals were sick was larger than the number of farmers who did not. There were no significant differences based on the sampling location.

**Figure-7:**
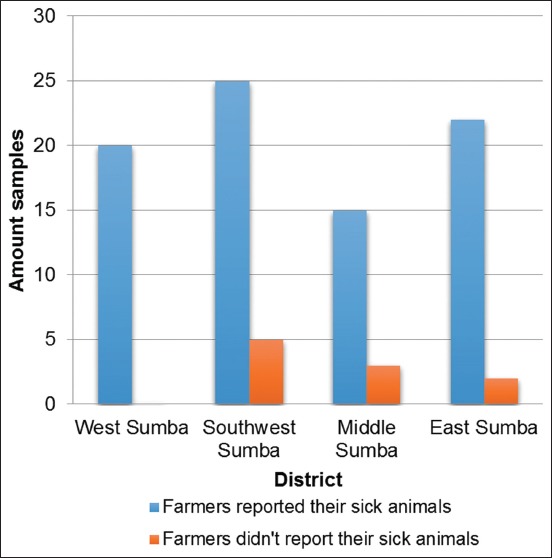
Histogram on the willingness of farmers to report their sick animals.

Among the farmers from the four districts, the farmers from Southwest Sumba had the highest level of awareness in terms of reporting the onset of the disease to a veterinarian (Figure-7). The horses raised by these farmers with a high awareness level in terms of reporting the disease onset had a lower seropositive surra percentage than those raised by farmers with a low awareness level.

## Discussion

The diagnostic technique of trypanosomiasis based on examining the clinical symptoms demonstrated a low level of sensitivity. It was allegedly because the horses were in an endemic area with a high incidence of chronic and subclinical infections. Claes *et al*. [7] reported that not all the horses that were subclinically infected revealed pathognomonic signs; a serological CATT was used because it had high specificity for *T*. *evansi* in horses and is cheap and easy to manage. Horses that are subclinically infected could be potential carriers of the *T. evansi* infection and could infect other animals that are in close contact with them. The positive results obtained via blood smear slides were allegedly influenced by the timing at which the sampling was conducted, which was in the rainy season [8]. Parasitological examinations carried out using Giemsa tinting of the blood smear slide was the gold standard in diagnosing *T. evansi*. However, it had low sensitivity. Elamin *et al*. [9] showed that the incidence of trypanosomiasis in the dry season was higher than in the rainy season. It was considered that this was a result of the increase in the activities of blood-sucking flies in this season. Tehseen *et al*. [10] reported that a CATT could also be used to aid in the diagnosis of *T. evansi* infections in camels. However, it could not provide information on whether the *T. evansi* infection was a current infection or a previous one [11]. In general, the percentage of positive CATT results in this study were higher than those generated via the tests carried out by Bill [12] on cattle in the East Nusa Tenggara Province. Furthermore, Mastra [13] studied the prevalence of *T. evansi* antibodies in horses on Sumbawa Island (Bima and Sumbawa Besar district) where horses had a higher mobility than those in the island of Sumba. It was believed that the life phase of mares influenced the susceptibility to *T. evansi*, while pregnancy and lactation could affect the horses’ immune system. Sumbria *et al*. [14] stated that mares were more easily infected by *T. evansi* than stallions. This might relate to pregnancy or lactation [15]. The results of our study on age as a risk factor (Figure 3) were similar to those of the study by Eyob and Matios [16] who stated that horses that were between 1 to 5 years old were more susceptible to the infection than those that were above 5 years or below 1 year old. This was believed to occur because young horses have an adequate level of antibodies needed to fight a *T. evansi* infection. Meanwhile, Tehseen *et al*. [17] stated that there were no differences in the ability to fight a *T. evansi* infection based on a horse’s age. In other words, horses of all ages could be infected. The farmers’ knowledge of surra was influenced by the surra outbreaks that occurred between 2010 and 2012, during which horse owners in Eastern Sumba began to learn about and understand the disease [18]. Highly knowledgeable farmers were the results of the government’s efforts to control the disease by disseminating useful information about surra. The level of a farmer’s knowledge generally correlates with their behavior [19]. Farmers who are highly knowledgeable will act proactively if they encounter a sick animal, such as reporting the animals to a veterinarian [20].

## Conclusion

The technique to diagnose surra based on examining the clinical symptoms and conducting parasitological examinations had a low sensitivity level. The CATT can be used to diagnose *T. evansi* infection in horses.

## Authors’ Contributions

The study was conceived, arranged, designed, and supervised by WN. MRKY carried out sampling and laboratory analysis. JP, SH, and MRKY analyzed and interpreted the data, while WN did the overall monitoring of the experiment and preparation of the manuscript. All authors read and approved the final manuscript.
